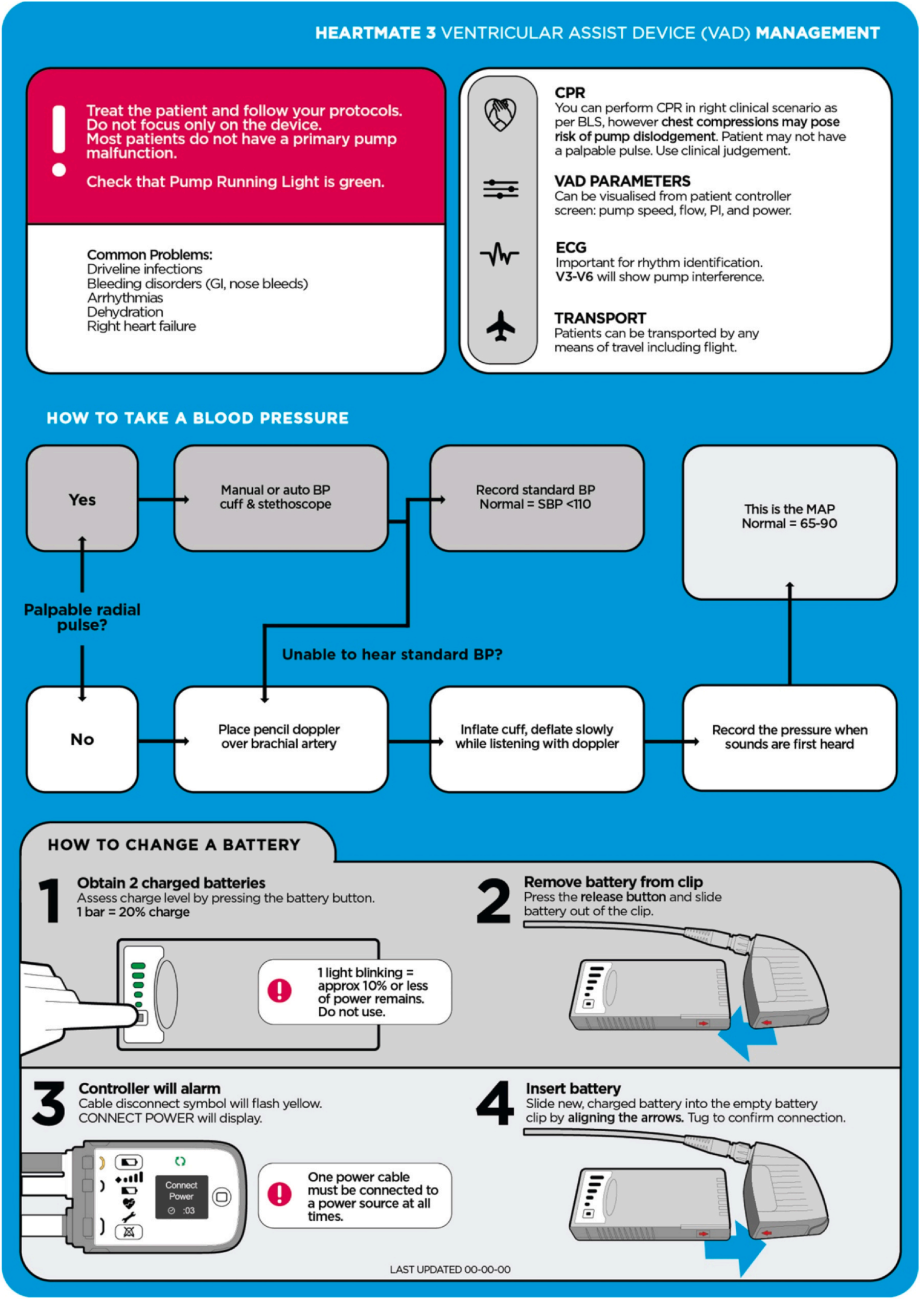# Co-designing emergency communication for patients with mechanical circulatory support devices

**DOI:** 10.1016/j.jhlto.2026.100522

**Published:** 2026-02-19

**Authors:** Leon Fitzpatrick, Genevieve Mosely, Thomas Davidson, Janelle McLean, Julia Rix, Cara Wrigley

**Affiliations:** a School of Architecture, Design and Planning, The University of Queensland, Brisbane, Australia; b Department of Cardiology, The Alfred, Melbourne, Australia

**Keywords:** mechanical circulatory support, co-design, visual communication tools, allied health collaboration, patient safety

## Abstract

The use of Mechanical Circulatory Support (MCS) devices is increasing globally, with patients often presenting to emergency departments (EDs) or encountering emergency medical services (EMS). Complex device protocols and limited non-specialist training can lead to treatment delays. This study describes a multi-stage, co-design process undertaken to develop an emergency communication poster for patients with a HeartMate 3 left ventricular assist device. The poster is intended to support rapid assessment and initial device management by non-specialist clinicians. The design process involved clinical observations, research, concept development, global evaluation, and iterative refinement. Eighteen international MCS practitioners contributed feedback on the prototype, informing refinements to information hierarchy, visual structure, and clinical clarity. The final outcomes include a rapid-reference poster and a set of MCS emergency communication design principles. Together, these outputs demonstrate how an iterative, context-sensitive design approach can enhance clinical communication and support shared understanding in time-critical MCS care across diverse healthcare settings.

## Introduction

The use of Mechanical Circulatory Support (MCS) devices is increasing globally, both as bridging therapy and as destination treatment.^1^, ^2^ Due to the technological advances of MCS devices, patient survival and quality of life continue to improve.^3^, ^4^, ^5^, ^6^

In addition to the technical improvements, patients’ survival, daily functioning, and quality of life depend, in part, upon the considerable support they receive before, during, and after MCS implantation.^7^, ^8^ MCS coordinators, such as Ventricular Assist Device (VAD) coordinators, are key providers of this support. These coordinators are typically highly specialized nurses with allied health and patient-facing expertise.^9^ They educate patients and family members, translate technical information, and liaise between patients, families, and clinicians across in- and out-patient settings.^9^, ^10^ MCS coordinators therefore support patients and their primary carers across the lifespan of MCS support.

Despite the advances in MCS devices, patients often experience adverse events and emergencies. Events such as bleeding,^11^ driveline infections,^12^, ^13^ cerebrovascular accidents,^14^, ^15^ and device malfunctions^16^ are common and often occur out of hospital.^14^, ^17^

As MCS patients transition to community-based care, clinical teams in emergency departments (EDs) and emergency medical services (EMS) are increasingly first responders for patient emergencies.^12^, ^18^ The current practice of EDs and EMS frequently relies on contacting a known MCS coordinator or transporting the patient to a specific hospital.^9^, ^18^, ^19^, ^20^ This reliance creates risks if coordinators are unavailable, or the patient presents to a different site.^9^, ^20^ In addition, communication in emergency settings is often improvised, with care processes relying on general clinical protocols that may not be standardized or tailored to MCS/VAD contexts.^18^, ^20^ MCS coordinators are therefore pivotal in bridging specialist knowledge and frontline response.^9^, ^21^

However, the complexity of MCS device protocols and the lack of specialist training among ED clinicians and EMS personnel present serious barriers to timely, informed care.^22^, ^23^, ^24^ These challenges often result in treatment delays and increased risk for patients.^18^, ^25^ As a result, there is a clear need for tools or processes that support rapid, safe, and standardized responses to MCS emergencies, regardless of coordinator availability.^18^, ^19^, ^20^, ^26^

The aim of this project was to design a visual communication tool that could function as a cognitive aid to support non-specialist personnel during MCS emergencies. In this paper, we discuss its design and preliminary evaluation, comprising feedback from an international survey with MCS coordinators.

## Methods and results

### Design process overview

**Figure 1:**
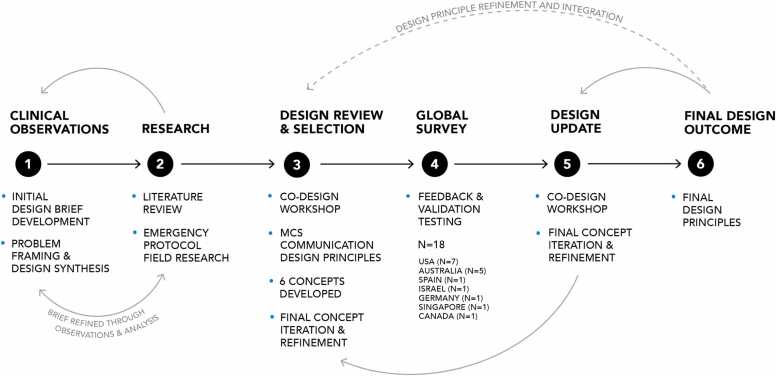
Co-Design Approach and Iterative Development of Final Design Outcome.

The design process consisted of 6 main stages: (1) clinical observations, (2) research, (3) design review and selection, (4) global survey, (5) design concept update, and (6) final design outcome (see Figure 1). The overall aim was to develop an emergency MCS protocol poster for the HeartMate 3 (HM3) left Ventricular Assist Device (VAD) that is intuitive, rapidly scannable, and suitable for use by non-specialist staff in time-critical scenarios.

The project originated from a clinical observation: ED staff reported difficulty determining immediate actions when managing MCS patients during shifts where trained MCS nurses were unavailable. This observation formed the basis of the design brief. The process involved collaboration between two MCS coordinators at a major Australian hospital (who generated the design brief), the design research team, and international MCS practitioners engaged through the ICCAC community. Contributors provided ongoing feedback on content accuracy, visual clarity, and clinical usability.

The design process was carried out within several constraints. Each hospital site has its own protocols, escalation pathways, and specification of essential information, meaning the poster needed to balance general applicability with device-specific accuracy. These variations shaped decisions about content inclusion, layout structure, and the degree of detail appropriate for emergency use. The study received ethical approval from The University of Queensland (2025/HE000048). In the following sections, we describe the design process and results of each stage.

### Stage 1 – clinical observations and design brief development

Stage 1 involved gathering clinical insights that informed the development of a clear and targeted design brief. Observations were drawn primarily from the extensive on-the-ground experience of two MCS coordinators at the Alfred Hospital in Melbourne, Australia, who had repeatedly encountered difficulties faced by ED staff when managing patients with a HM3 device. These insights accumulated over years of clinical practice, after-hours call-outs, and repeated interactions with ED teams during unplanned patient presentations.

Clinicians identified several persistent issues with existing MCS communication resources. Although comprehensive device manuals and ICCAC-produced reference documents were available, they were lengthy, text-heavy, and difficult to navigate during time-critical scenarios. Staff reported that essential information was buried within large documents, not visually prioritized, and required prior familiarity to use effectively. As a result, non-specialist staff often struggled to quickly access the steps required to stabilize an MCS patient in an emergency.

The MCS coordinators described receiving frequent after-hours calls from ED clinicians seeking immediate guidance when an HM3 patient presented unexpectedly. Similar challenges were confirmed through informal conversations with industry stakeholders, including the Vice President of Abbott (HeartMate 3), who noted that this issue also occurred internationally. The problem was compounded in the Australian context, where patients often return to suburban or regional areas far removed from major VAD centers, increasing the likelihood that first-response clinicians may have limited MCS experience.

Across these clinical insights, a consistent problem emerged: frontline clinicians lacked a rapid, trustworthy, and easy-to-use communication aid that distilled the essential steps for initial HM3 emergency management. These insights were synthesised into a design brief that specified the poster’s purpose, core content requirements, intended users, and operational context, forming the foundation for the subsequent design stages.

These practice-based insights were progressively refined through follow-up conversations with the coordinators. This process produced a clear definition of the problem and a shared understanding of the situational pressures faced by clinicians. The design team synthesised these insights into a structured design brief that articulated the following.

• *The purpose*: to create a rapid, intuitive communication aid to support initial management of HM3 emergencies.
• *The functional requirements*: essential information that needed to be immediately scannable, clinically accurate, and usable by non-specialist staff.
• *The intended environments*: primarily emergency departments and other acute care contexts where rapid decision-making is required.
• *The contextual constraints*: variation in local protocols, differing staff expertise, and the need for a tool that could be understood without specialist training.

This design brief provided the foundation for the subsequent research, concept generation, and evaluation stages.

### Stage 2 – research (literature, desktop, field)

Stage 2 involved literature, desktop, and field research to understand existing approaches to emergency communication design and to identify opportunities relevant to MCS emergencies. The literature and desktop reviews confirmed the absence of established principles or guidelines for the design of emergency MCS communication tools.

To address this gap, the design team undertook field research to examine a diverse set of emergency protocols, including medical, fire, rescue, and public safety materials, to identify transferable communication strategies. The analysis focused on: (1) *context of use*, which included where protocols were located, whether they appeared as standalone artifacts or alongside other materials, how visible and easy to locate they were within the environment, and whether they were legible or immediately identifiable from a distance; and (2) *design characteristics*, which examined the strategic use of color, consistency of typography, layout and graphics, text conciseness, information hierarchy, balance between density and breathing space, and clarity of graphic elements.^27^ Attention was paid to whether protocols enabled rapid comprehension and step-by-step following during time-sensitive events.^28^

These analytical criteria guided a series of team-based critiques. Using printed artifacts and photographs of protocols, the design team conducted iterative whiteboard analyses to document observations, identify communication breakdowns, and capture high-value communication strategies.^27^ Findings were grouped, themed, consolidated, and refined into an initial longlist of potential design principles. This list was further synthesised into six primary communication design principles that were ordered by their perceived importance in supporting rapid, safe, and intuitive emergency response, as follows.

1. **Hierarchy –** Clear sequencing and ordering of information to support rapid eye-fall and logical flow between elements. *Considerations: prioritizing content by urgency and importance; structuring information to guide immediate action.*
2. **Composition** – Effective arrangement and proportional balance between elements. *Considerations: grouping related components, maintaining spatial clarity, and using modular structures where appropriate.*
3. **Color** – Strategic use of hue, contrast, and saturation. *Considerations: using color to emphasize critical information or reinforce visual grouping rather than as decoration.*
4. **Continuity** – Consistency in graphical conventions across elements or formats. *Considerations: maintaining coherent typographic choices, iconography, line weights, and color systems.*
5. **Legibility** – Immediate readability of text and icons at the point of use. *Considerations: applying human factors guidance on type size and spacing, and testing designs at full scale.*
6. **Scale** – Suitability of design for the intended display format. *Considerations: ensuring clarity across print and screen contexts, accounting for viewing distance, and maintaining fidelity at different resolutions.*

These principles formed the foundation for the generation and assessment of early visual concepts in Stage 3.

### Stage 3 – concept generation and internal review

The design team generated six discrete, intentionally divergent concept directions through an iterative process that combined broad ideation with targeted refinement. Divergence at this stage was purposeful: different designers respond to a brief in different ways, and multiple concepts enabled broader exploration of visual strategies, communication structures, and interpretations of the design principles identified in Stage 2.^29^ Producing multiple concepts also enabled more effective comparison and discussion with the MCS coordinators, who were the primary decision-makers for clinical accuracy and contextual fit.

Each concept was reviewed in a structured session with the MCS coordinators and the design team. Coordinators marked up concepts, highlighting elements that best aligned with clinical practice, and that most effectively addressed the brief. Their feedback focused on text accuracy and clarity, diagram usefulness and legibility, effectiveness of graphic communication, and the overall hierarchy of information—particularly whether essential steps for early emergency management were foregrounded.

**Figure 2:**
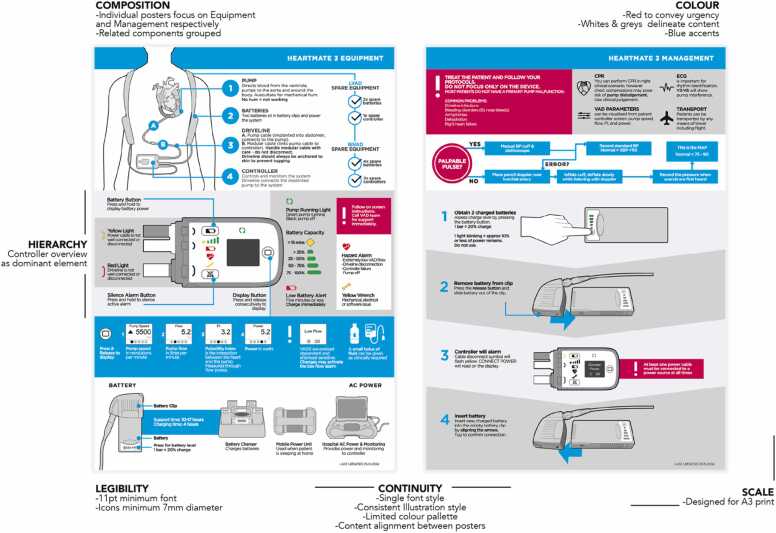
Prototype HM3 VAD poster used for evaluation in the global survey with annotated design principles. Note that the annotated design principles were not shown to participants.

Through this collaborative evaluation, a single “hero” concept was selected for further development. This concept underwent several rounds of refinement, with substantial changes made to improve its clarity, accuracy, and usability. Major revisions included adjustments to proportions and layout hierarchy, reductions in text density to support rapid scanning, refinements to the color scheme, and significant redevelopment of the controller overview section to ensure precise communication of device functions, lights, and alerts. Across these iterations, the emerging communication design principles from Stage 2 were progressively narrowed, consolidated, and embedded into the evolving visual design, producing a coherent and usable poster prototype for evaluation in Stage 4 (see Figure 2).

### Stage 4 – global survey evaluation

A global survey evaluation was used to capture practitioner insights on the prototype HeartMate 3 (HM3) VAD poster developed in Stage 3. Specialist VAD practitioners were recruited via email and asked to complete a 17-item survey. The survey assessed: (1) the poster’s goals and relevance (9 items); (2) the effectiveness of design features including legibility, grouping, sequencing, illustrations, color, and text-graphic balance (6 items); and (3) opportunities for improvement (2 open-ended questions). Likert-type items were scored from 1 (strongly disagree) to 4 (strongly agree). Quantitative responses were analysed descriptively (median and IQR), and qualitative comments from the two open-ended questions were thematically reviewed to identify common patterns and areas for refinement.

Eighteen specialist VAD practitioners completed the survey. Respondents were from the USA (n=7), Australia (n=5), Spain (n=1), Israel (n=1), Germany (n=1), Singapore (n=1), and Canada (n=1). Participants included MCS/VAD coordinators (n=10), clinical nurse specialists (n=2), clinical nurse consultants (n=2), specialist transplant coordinators (n=2), and a specialist nurse (n=1). One participant did not provide their role or work location. Most participants had experience with the HeartMate 3 (n=16), with additional experience spanning multiple device types, including HeartMate 2, HVAD, Impella, Centrimag, EXCOR, and other VADs.

#### Perceived effectiveness of the poster’s relevance and goals

**Figure 3:**
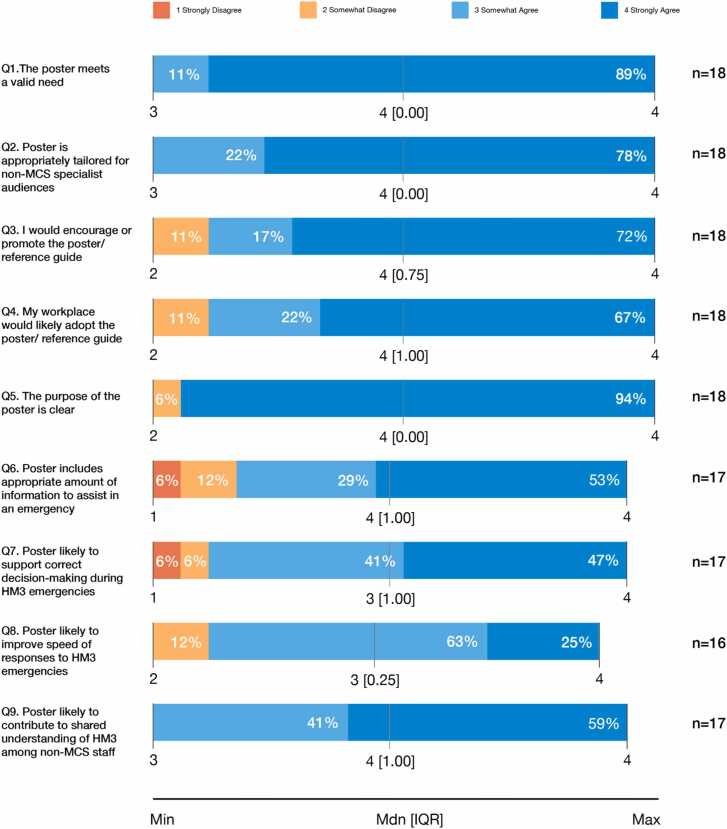
VAD Practitioners’ Perceptions about the Poster’s Clarity, Content, and Usability.

VAD practitioners rated the poster positively across all evaluated features, with the majority indicating strong agreement for most items (see Figure 3). Almost 90% of respondents strongly agreed that the poster meets a valid need and that its purpose is clear, while 78% felt it was appropriately tailored for non-MCS specialist audiences. Most participants reported they would encourage or promote the poster and felt their workplace would likely adopt it. Items related to emergency applicability showed slightly more variability with some participants indicating that the poster may not fully support correct decision-making or speed of response during HM3 emergencies. Overall, responses from 82–100% of participants indicated agreement to questions about the poster’s goals, supporting its validity and relevance across international MCS/VAD practice settings.

Participants’ qualitative comments confirmed the poster’s clarity and ability to translate complex HM3 information into accessible terms for non-specialist audiences. For example, one participant stated,*“it does a good job of breaking down the information into laymen terms so non-MCS clinicians can better understand the functionality of the equipment and what is concern worthy versus less critical.”* Feedback also highlighted limitations for acute emergency use, with some participants suggesting that the poster might serve best as a reference tool following practical education or training sessions. For example, one participant stated, *“In emergency situations involving a VAD patient, I don’t think this poster will be a leading resource in managing these situations.”* Further suggestions included introducing mnemonics or more structured step-by-step emergency guidance.

#### Perceived effectiveness of the poster’s visual layout and design features

**Figure 4:**
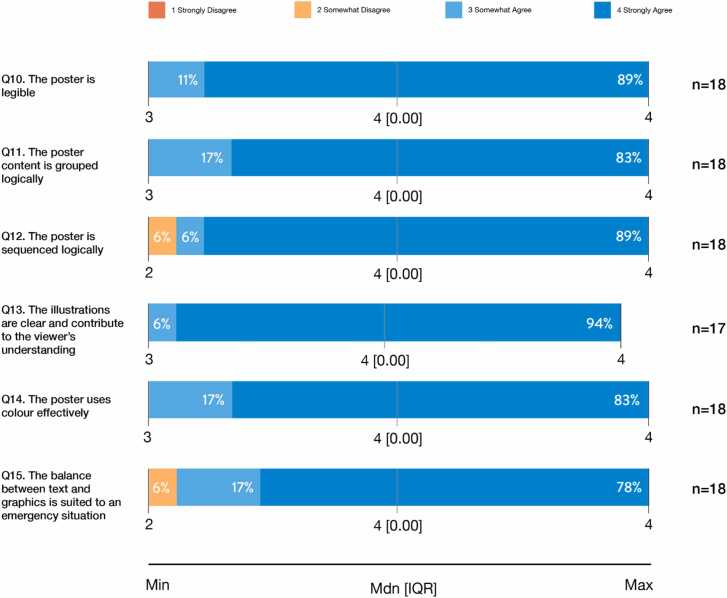
VAD Practitioners’ Perceptions of the Poster’s Visual Layout and Design Features.

Participants rated highly the poster’s design features and visual layout, with strong agreement on legibility, content organization, and visual clarity (see Figure 4). While all features received positive ratings, the balance between text and graphics for emergency reference showed slightly more variability, reflecting some differences in perceived usability under time-sensitive conditions. Overall, 95–100% of participants agreed that the poster’s design features effectively supported understanding and usability.

#### Suggestions for improvement

Qualitative feedback from the specialist VAD practitioners highlighted opportunities to enhance the posters’ clarity, usability, and applicability in emergencies. Participants suggested emphasizing stepwise emergency management, enlarging critical guidance (e.g., blood pressure targets), and clarifying device operations. For example, one participant noted, *“There is no information about how to change a controller, driveline damage, modular cable, or other components.”* Additional suggestions included references to training patients and caregivers. For example, one participant noted, “*Some reference to the patient and caregiver being trained in the device would be good*”, while another recommended, *“more live scenario step-by-step assessment tool.”* Suggested layout improvements included reordering device components (e.g., pump, driveline, controller, batteries), adding subheadings (*“Provide a subheading in each section, this will help staff unfamiliar with the device”)*, and color coding procedural steps (*“Use a different color for the steps in taking a doppler reading and a cuff reading”*). While several respondents noted the poster was already clear and visually appealing (*“it looks great and is super clean”*), these refinements were identified as ways to maximize its effectiveness as a rapid-reference tool for non-specialist clinicians, particularly in time-sensitive situations.

### Stage 5– integration and design refinement

**Figure 5:**
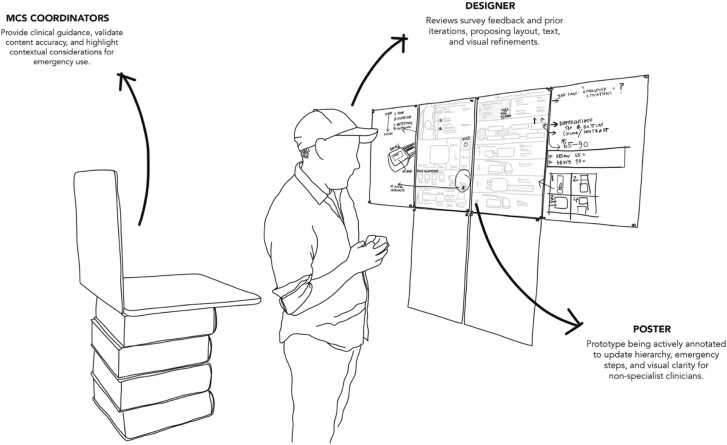
Collaborative annotation of the HM3 poster by the design team and MCS coordinators, integrating survey insights and iterative refinements.

Global survey responses from Stage 4 were collated and analysed using a structured thematic coding process^30^ that focused on clarity, usability, clinical accuracy, and visual communication. The design team then workshopped the coded feedback with MCS coordinators in a series of collaborative sessions (see Figure 5), interpreting feedback through a clinical lens, determining which issues required design action, and highlighting contextual differences between health systems.

From here, insights were translated into a set of targeted design refinements. The team systematically reviewed the poster’s layout, language, and graphics, making adjustments where the survey indicated confusion, ambiguity, or opportunities to strengthen emergency usability. In parallel, the preliminary design principles were reviewed, consolidated, and updated to reflect the iterative learning gained from concept development, internal critiques, and global feedback. This stage concluded with an updated prototype and a revised set of design principles.

### Stage 6 – final design outcome

In the final stage, the design team produced the completed HM3 emergency poster (see Appendix A) and finalized the associated MCS communication design principles. The Stage 5 prototype was translated into a production-ready version through a series of internal checks for visual consistency, clinical accuracy, and usability. MCS coordinators verified device-specific instructions, emergency action sequencing, and terminology. The poster was then prepared for multi-context clinical use, including versions for different print sizes and integration into training materials.

## Discussion

The evaluation of the HM3 VAD emergency poster highlights its strengths and limitations as a communication tool for non-specialist staff and demonstrates the benefits of using a co-design approach. MCS coordinators and clinicians consistently valued the poster’s clarity, accessibility, and visual design, confirming its role as a fit-for-purpose aid that translates complex device information into understandable terms. High levels of endorsement suggest the poster addresses a recognized need and has potential for adoption across clinical settings. At the same time, variability in perceptions of its utility during high-pressure situations points to important considerations for further refinement. Feedback emphasized the balance between accessibility and clinical accuracy, the need for integration into training and simulation, and the importance of adaptability across diverse health systems. Overall, these findings position the poster as an environmental communication artifact that supports shared understanding, while highlighting the limits of static tools when used in isolation during acute emergencies.

The findings support previous research by highlighting the challenges of balancing clarity with clinical accuracy when designing cognitive aids to support staff effectively during clinical emergencies.^28^, ^31^, ^32^ Participants appreciated the poster’s ability to translate complex HM3 VAD information into accessible terms, noting that this would likely make it easier for non-specialist staff to engage with the content. At the same time, some respondents expressed concern that certain simplifications, such as guidance on fluid boluses or MAP calculations, could risk misinterpretation in urgent situations. This tension was navigated throughout the design process through iterative concept development, review, and refinement with MCS specialists. The need to simplify visual information without compromising clinical intent underscores the importance of involving MCS specialists in a co-design approach. Developing tools with MCS specialists ensures that the tools remain understandable, provide clear and reliable guidance, and minimize the potential for errors that can compromise safety in high-stakes clinical contexts.^28^, ^29^, ^31^, ^33^

The study further highlights factors that influence the adoption and broader applicability of environmental supports in clinical practice. Participants strongly endorsed the poster, indicating personal willingness to use it and confidence that their workplaces would adopt it, reflecting high perceived validity and utility. However, successful uptake depends on more than the presence of a poster alone. Embedding these resources within workplace routines, structured training programs, and simulation exercises can enhance their relevance and impact, fostering familiarity, confidence, and preparedness across multidisciplinary teams.^28^, ^34^, ^35^, ^36^, ^37^ Additionally, feedback from international VAD specialists suggests that the poster’s design principles have wide applicability. However, local adaptation is necessary to account for device-specific details, language translation, and variations in health system structures.^18^, ^20^, ^21^, ^26^ Achieving the right balance between standardization and contextual flexibility reflects a key benefit of using a co-design approach.

Finally, the study identifies important limitations and directions for future work. While participants supported the poster’s clarity and design, perceptions varied regarding its applicability in true emergencies. The results are also constrained by the sample size and by the fact that the poster was evaluated outside of representative acute scenarios by specialist staff who were familiar with HM3 devices. As such, perceptions of usefulness cannot be assumed to equate to performance under real emergency conditions. Future research should focus on iterative refinement of the poster, incorporating feedback from diverse and non-specialist clinical users, integrating it with standard processes for patient emergencies,^14^, ^38^ and testing its effectiveness within simulation-based emergency training.^28^, ^31^, ^33^ Integrating the poster with other communication tools and multimodal supports, such as smartphone applications^39^ or pocket reference cards,^40^ could further enhance its utility. These future directions align with a continuation of the design process, rather than a discrete evaluation endpoint. In this way, emergency communication tools can be designed to provide a clear reference while also contributing meaningfully to staff decision-making, shared understanding, and, in turn, patient safety in high-pressure MCS emergencies.

## Implications for allied health practice

The findings emphasize the value of designing emergency communication aids that are accurate, clear, and tailored to the contexts in which allied health professionals engage with MCS patients. Prioritizing non-specialist use throughout the design process, demonstrates how co-design with MCS coordinators, clinicians, and allied health staff can support both clinical accuracy and trust in how visual communication tools are used in practice. Communication aids must also remain adaptable to device-specific requirements, local workflows, and varied experience levels.

Embedding tools such as the HM3 poster within emergency departments has the potential to strengthen shared understanding and provide a stable point of reference when MCS specialists are not available. Rather than replacing established emergency processes, such aids are designed to reinforce key actions and concepts under conditions of uncertainty, thereby supporting rapid, confident engagement with unfamiliar technology.

International feedback highlights the opportunity for broader collaboration in developing and validating emergency MCS resources. Shared, context-derived communication design principles can be scaled across health systems, while local adaptation maintains relevance and safety. Integrating these tools into training, simulation, and interdisciplinary education further extends their impact, enhancing workforce preparedness and professional development across allied health and non-specialist teams.

## Declaration of Competing Interest

The authors declare the following financial interests/personal relationships which may be considered as potential competing interests: Cara Wrigley reports financial support was provided by Australian Government Department of Health Disability and Ageing. Janelle McLean reports a relationship with Abbott Medical that includes: consulting or advisory and speaking and lecture fees. If there are other authors, they declare that they have no known competing financial interests or personal relationships that could have appeared to influence the work reported in this paper.

## Figures and Tables

**Figure:**
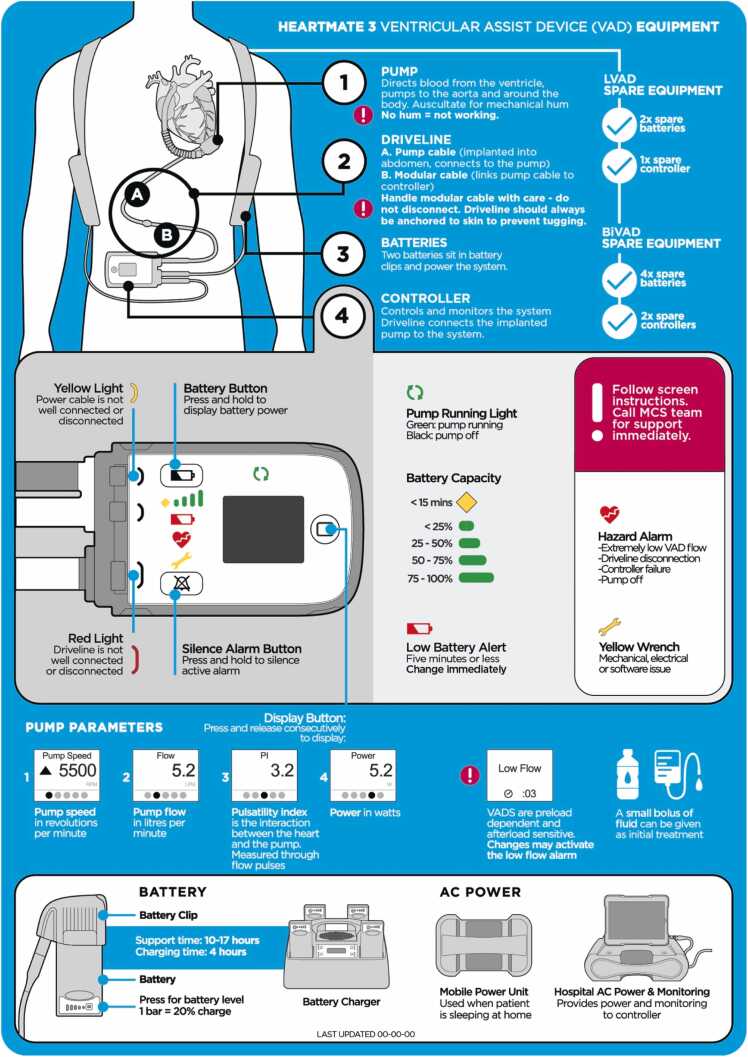


**Figure:**